# A community‐partnered process for construct & measure development: The 3Rs: Reading, Racial equity, & Relationships

**DOI:** 10.1002/ajcp.70010

**Published:** 2025-08-27

**Authors:** Shannon B. Wanless, Meghan C. Orman, Shallegra Moye, Caitlin F. Spear

**Affiliations:** ^1^ University Center for Social & Urban Research University of Pittsburgh Pittsburgh Pennsylvania USA; ^2^ Office of Child Development University of Pittsburgh Pittsburgh Pennsylvania USA; ^3^ Present address: College of Human Ecology Cornell University Ithaca New York USA

**Keywords:** community‐based participatory research, community‐partnered research, literacy, psychometrics, racial equity

## Abstract

This paper describes a 3‐year community–partnered research initiative focused on advancing early reading, racial equity, and relationships—collectively known as the 3Rs Initiative. The project brought together researchers and community members committed to ensuring that all adults in the county embody a shared “3Rs mindset” to better support literacy development for children in kindergarten through third grade. In Study 1, researchers and community members (The 3Rs team) used thematic analysis of interviews, meeting notes, and group activity and discussion artifacts with community stakeholders to construct and validate a definition of a 3Rs mindset. In Study 2, the 3Rs team created a 36‐item scale that could assess an adult's 3Rs mindset. The scale demonstrated excellent content validity, response process, and associative validity. Findings from both studies suggest that community‐partnered measure development can be achieved through collaboration, honoring of multiple perspectives, and elevating community partners as “experiential experts” (El Mallah, 2024, p. 984). Measures resulting from such processes have the benefit of community‐defined cultural specificity and strong content validity and can be leveraged towards social change.

## Highlights

- Researchers and community members co‐defined an adult mindset for a new 3Rs initiative.
- A 36‐item scale was created to assess a 3Rs adult mindset and it had strong validity.
- This process reflected collaboration, honoring multiple perspectives, and positive social change.

Social determinants of health, such as early literacy, are receiving national attention because of their long‐term impacts on individual and community thriving. Specifically, early literacy was named as a national priority in *Health People 2030* (Office of Disease Prevention and Health Promotion, n.d.) because it predicts later well‐being, educational attainment, lifelong earnings, and likelihood of incarceration (Annie & Casey Foundation, 2011; National Assessment of Adult Literacy, 2016). Children in the U.S., however, are struggling to learn to read. In 2022, only 33% of U.S. 4th grade students were reading at or above a proficient level according to the National Assessment of Educational Progress (n.d.). This issue has stark implications for equity, as only 17% of 4th grade Black students scored at or above proficient compared with 42% of their White peers, a disparity that has persisted for decades (National Association of Educational Progress, n.d.).

Racial disparities in reading are widespread and persistent, and they have dire long‐term impacts, including high school achievement and graduation rates, long‐term economic prosperity, and even incarceration rates (e.g., Annie & Casey Foundation, 2011; Foorman et al., 2018; National Assessment of Adult Literacy, 2016). Racial disparities in reading outcomes are the result of opportunity gaps—unjust policies, practices, and procedures that result in the majority of Black students receiving unequal access to opportunities that lead to positive reading outcomes (e.g., inequitable school funding, teacher training issues, inequitable access to high quality early childhood opportunities or challenging curriculum; Gorski, 2019; Milner, 2012; Milner, 2025; Whittingham & Hoffman, 2024). Despite this well‐established understanding, traditional remediation approaches to addressing lagging reading scores may be effective at improving specific skills but have not been effective in reducing overall disparities, which may be in part due to the fact that most evidence‐based reading interventions rarely focus on Black children or respond to the role of race in reading disparities at all (Milner, 2020).

Interactions with adults from many areas of a child's literacy ecosystem (e.g., school, home, community organizations) impact literacy development, and it is ideal when those interactions are warm, responsive, informed by the science of reading, and promote equity (Milner, 2021). Coordinated, community‐wide efforts targeting different areas of the literacy ecosystem may be especially effective in promoting early literacy development, particularly for vulnerable populations (Akiva et al., 2017; Peifer & Perez, 2011). To support such efforts, it is essential to understand the beliefs and values—that is, mindsets—that adults bring to their literacy interactions with children. There are large bodies of research exploring the impacts of teacher beliefs and mindsets on practice (e.g., Guskey, 2002; Laine & Tirri, 2023; Pajares, 1992), based on the well‐established theories that teachers play critical roles in shaping student experiences, and that teachers' attitudes and beliefs about students, learning, and instruction will shape their practice (e.g., Buehl & Beck, 2015; Schachter et al., 2016; Pianta et al., 2014; Kennedy, 2016). Considering this, a countywide effort to improve literacy was created to help adults develop a mindset that incorporated racial equity and responsivity into their literacy interactions with children in every context. Named the 3Rs (Reading, Racial Equity, & Relationships), this community‐partnered initiative sought to address racial disparities in literacy (kindergarten – third grade) by advancing racially equitable literacy practices in homes, schools, and community learning spaces.

**Figure 1:**
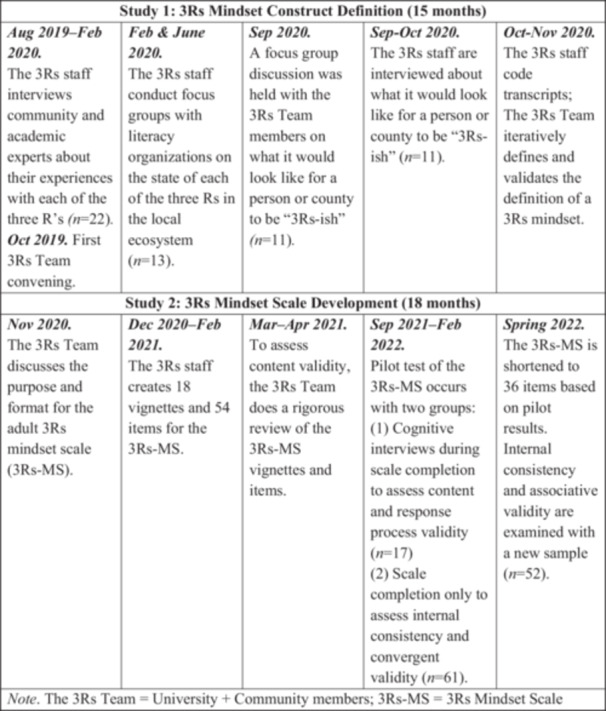
Timeline for the 3Rs mindset construct definition and scale development. Note. The 3Rs Team = University + Community members; 3Rs‐MS = 3Rs Mindset Scale.

In this paper, we describe the process the 3Rs used to cocreate a definition of an adult “3Rs mindset” that integrates Reading, Racial Equity, and Relationships in all literacy interactions with children (Study 1), and cocreate a measure of it (Study 2). This 3‐year, iterative, mixed methods process (Figure 1) was grounded in key tenets of community‐based participatory research (CBPR) including collaboration, honoring multiple perspectives, and positive social change. Our goals were to elevate community voice and history, and be informed by research literature and best practices in measure development. This paper is an illustration of how CBPR tenets can help to frame community‐university partnerships that aim to integrate the strengths of both partners to advance positive change in a region (Ohmer et al., 2022). In this paper, the reader can expect to find a university‐community partnered application of CBPR to develop a definition and measure that would lay the foundation for a countywide effort to eliminate racial disparities in early literacy.

## SITUATING THE 3RS: READING, RACIAL EQUITY, AND RELATIONSHIPS

From the beginning of this community‐university partnership, it was evident that racial disparities played a central role in the literacy ecosystem's history, in families' lived experiences, and as a defining feature of existing local literacy data. In the county, there was a 40‐point gap between White and Black third graders' scores on state literacy test before the COVID‐19 pandemic, which widened to 50 points in 2021. At that point, 77% of White students passed state tests compared to 28% of Black students (Pennsylvania Department of Education, 2023). Although there are complex systemic issues (e.g., school funding, segregation, access to high‐quality early learning experiences), that lead to racial disparities in literacy, these systems are made of individuals (e.g., teachers, administrators, policymakers). Individuals' practices and decisions reflect their individual ideologies (Milner, 2021). When individuals in a literacy ecosystem understand how they can disrupt racial inequities, their daily interactions in systems begin to reflect that.

As the 3Rs Initiative began, critical scholars called for individuals to learn how to disrupt racial inequities in literacy interventions (Hammond, 2021; Muhammad, 2022), and so was our state Department of Education in their culturally responsive teaching framework (Pennsylvania Department of Education, 2022). As a community‐engaged initiative, listening to current and historical community voices calling for attention to racial equity in literacy, was essential. In 2019, a board member from a local school district released a memo outlining explicit concerns over racial disparities in literacy development in the district (Udin, 2019). Later that year, the same district outlined an equity plan to address racial disparities in the district. Previous efforts to address racial disparities in literacy outcomes existed in the community. These plans had a historical lineage including a literacy project called the Triple M (Multicultural, Multiethnic, Multiracial) Program (1982−1992).

The Triple M Program was a collaborative effort among administrators, teachers, parents, and community members to address opportunity gaps between Black and White middle school students by equipping adults to address race and culture in the classroom (Gottfredson et al., 1992). The program ran for 3 years and saw some success, but literacy gaps persisted. A significant challenge was that the construct, “Triple M” had not been operationalized and there was no way to measure it with “agreed‐upon indicators” (Gottfredson et al., 1992; p. 87). The 3Rs Initiative listened to the current and historical context of its community and broader academic field, and sought to incorporate racial equity and responsive relationships into adults' literacy interactions with children. It was clear that developing an agreed‐upon measure to assess the efficacy of the initiative would be a priority.

**Figure 2:**
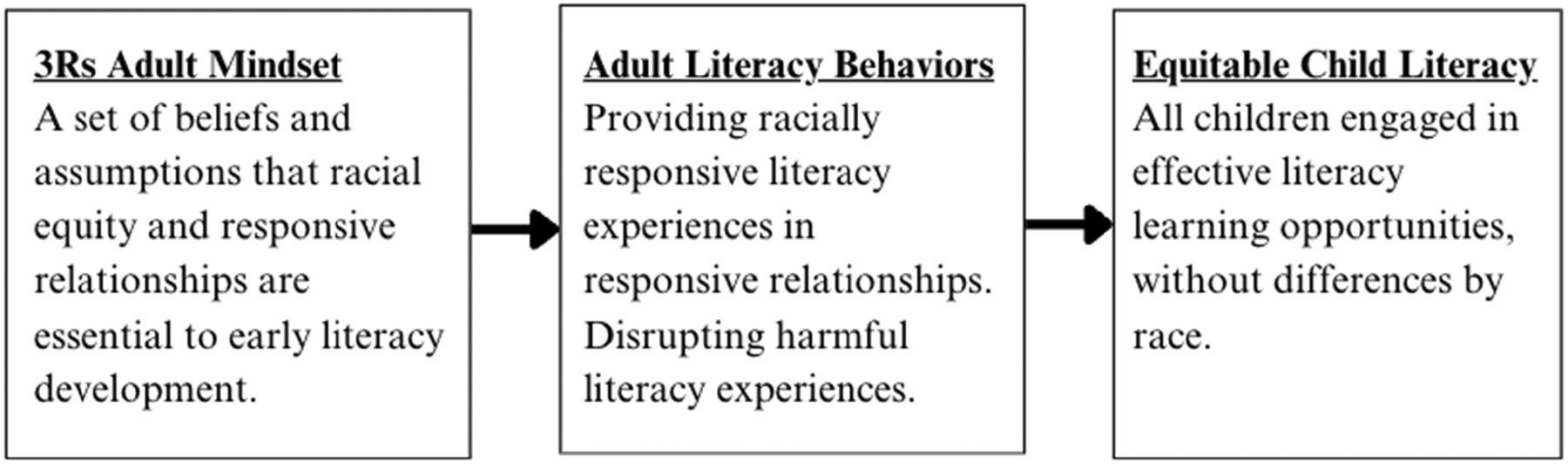
Proposed theory of change: Adult mindsets and child literacy.

The concept of an adult mindset that could be operationalized and measured became central to the 3Rs Initiative. Buchanan and Greig (2021) define mindsets as “deeply held beliefs and assumptions that we create about who we are and how the universe works” (p. 493), and note that it is the “capacity to shift mindsets that can transform the possibilities we are able to see and actualize in our lives and in our communities” (p. 494). In education, mindsets are associated with adults' implicit attitudes and beliefs about how children learn (Dweck, 2006) and have been shown to impact educator behavior (DeLuca et al., 2019; Kouzes & Posner, 2019) and child outcomes (Kroeper et al., 2022; Yeager et al., 2022). For example, a mindset that views Black children as troublemakers or underachievers may impact an educators' perception of a Black child's skills, abilities, and ambitions (James, 2012), potentially leading to the educator having significantly lower expectations for that child (Gershenson et al., 2016). On the other hand, a mindset that views Black children as having inherent strengths may opt for literacy practices that build on those strengths, potentially leading to improved reading outcomes (Acosta & Duggins, 2018). Cultivating a mindset that views racial equity and responsive relationships as integral to literacy development—i.e., a 3Rs mindset—is essential to shaping adults' interactions with children to be more equitable and responsive (see Figure 2).

The 3Rs mindset includes understanding the way racial inequities are created in systems and the importance of relationships as a context for engaged learning. Scholars have described ways that adults shift their literacy interactions with children when developing a racially‐informed mindset. Examples include (1) countering racially harmful messages in their curriculum (e.g., Whittingham & Hoffman, 2024), (2) choosing racially affirming books to engage children (e.g., Spear et al., 2023; Wanless, et al., 2022; White & Wanless, 2019), (3) using literacy approaches that build children's social awareness (e.g., Kazmarczyk et al., 2019), and (4) engaging in culturally responsive literature practices that prioritize high expectations and the cognitive development of students from marginalized backgrounds (e.g., Hammond, 2021). Thus, the 3Rs Initiative aimed to cultivate a 3Rs mindset to inform adults' use of curriculum, book selection, home activities, community‐based programming, and leadership decisions across the ecosystem and eliminate racial disparities in literacy.

### Theoretical framework

The 3Rs initiative is grounded in a bioecological systems perspective that views learning as shaped by dynamic interactions between individuals and their environments (Bronfenbrenner, 1979; Lee et al., 2023). Drawing from the learning ecosystem framework (Falk et al., 2015), we conceptualize literacy development as occurring not only in formal educational settings, but also within a broader constellation of families, communities, peer relationships, and informal learning spaces (Orman & Wanless, 2024). This aligns with Bronfenbrenner's (1979) bioecological model, which emphasizes the importance of nested systems—microsystems, mesosystems, exosystems, and macrosystems—in shaping child development. These theoretical approaches center relationships, place‐based context, and systemic influences, making them particularly well‐suited to community‐engaged efforts that seek to advance equity in learning (Akiva et al., 2023; Sunsern & Lawang, 2019). By recognizing communities as integral parts of the learning ecosystem, we approach our collaboration not simply as a strategy for data collection, but as a core epistemological stance. This orientation aligns closely with engaged approaches to research—like community‐based participatory research (CBPR)—which value shared knowledge production, co‐ownership of tools, and honoring local expertise (Israel et al., 2019).

## COMMUNITY‐PARTNERED CONSTRUCT AND MEASURE DEVELOPMENT

CBPR has a long history in community psychology as a method for co‐creating solutions to systemic inequities in areas such as public health, nursing, and medicine (Rodriguez Espinosa & Verney, 2021; Wallerstein, 2021). CBPR is a paradigm that engages communities in the design, conduct, and dissemination of research for social change (Center for Social Justice and Community Action, 2022). This approach emphasizes the importance of developing enduring, equitable relationships that connect researchers, practitioners, and community stakeholders throughout all stages of research to address real‐world problems (Israel et al., 2005; Penuel et al., 2020). Central to CBPR is acknowledging the multifaceted nature of community identity, encouraging the inclusion of diverse groups, and affirming their lived experiences and strengths (Israel et al., 1998; Miller et al., 2018). As such, CBPR offers a persuasive—and perhaps underutilized—framework for engaging community expertise in the design of equity‐centered educational tools and interventions, including those focused on literacy and reading equity (Gonzalez & Trickett, 2014; Parker et al., 2020).

In the present study, we aimed to apply these CBPR tenets to define and measure the 3Rs mindset construct. Constructs and measures are shaped by the epistemological, cultural, and political beliefs and values of those who create them (Malorni et al., 2023). Sharing power and following the lead of community members whose lives are impacted by a social issue can make it more likely that constructs and measures reflect the epistemological and cultural perspectives and values of those closest to the issue (Center for Social Justice and Community Action, 2022). CBPR's tenets applied to construct and measure development have been less frequently discussed in the literature. Here, we describe our experience with CBPR in defining and measuring a 3Rs mindset.

### Applying CBPR principles to measure development

CBPR is defined by three principal tenets: (1) collaboration, (2) honoring multiple perspectives, and (3) positive social change (Strand et al., 2003). Collaboration is characterized by mutual trust and respect, empathy and understanding, clear communication, and shared power. Collaboration is essential throughout the entire process—not just during pilot phases—to ensure construct validity (Arora et al., 2015). Collaboration also supports construct equivalence (i.e., ensuring the construct researchers think they are measuring aligns with community members' lived experiences), the contextual relevance of scale items and language, and the negotiation of community members' and researchers' epistemologies and worldviews (Gonzalez & Trickett, 2014). It often occurs across identities, requires vigilance against institutionalized and internalized racism, and benefits from cultural humility (Minkler, 2004).

Honoring multiple perspectives is characterized by co‐learning among researchers and community members and validating different types of knowledge and ways of knowing. Without broadening the idea of what counts as knowledge and incorporating community expertise into construct construction, the content validity and cultural appropriateness of measures of these constructs (e.g., 3Rs mindset) are limited.

Positive social change in CBPR means focusing on a goal highlighted by the community (Center for Social Justice and Community Action, 2022), which is often complex and persistent, like inequitable access to quality literacy experiences. It often means long‐term, iterative research that is attentive to root causes (Israel et al., 1998; Strand et al., 2003). In the present study, we sought to define a construct (e.g., 3Rs mindset) that could be contributing to a social issue (e.g., low literacy rates) and develop ways of measuring it to support long‐term intervention.

## OVERVIEW AND CONTEXT OF STUDY 1 & STUDY 2

The 3Rs Initiative was a community‐partnered effort that was mutually beneficial and that respected the community that had a long history of providing children with literacy experiences. This approach matched the legacy of the 3Rs' anchor organization, The Office of Child Development (Groark & McCall, 2018) and their parent study, The Pittsburgh Study (TPS; Ettinger et al., 2022), at the University of Pittsburgh. The 3Rs research team turned to the community to better understand their experiences with each of the 3Rs and understand opportunity gaps in early literacy. As ideas emerged, the 3Rs research team began referring to the collective vision as a county where adults were more “3Rs‐ish.” The following studies are the 3‐year, iterative, mixed methods process to develop that construct (3Rs mindset; Study 1) and measure (Study 2; Figure 1).

Across both studies, the 3Rs Team—up to 25 people—was comprised of the 3Rs university staff and a cohort of community members who constituted at least 50% of the committee (Ettinger et al., 2022). Community members had a vested interest in early reading and racial equity, and included (often with overlapping roles) parents, educators, school leaders, community organizers, and organizational leaders. The 3Rs team considered the community members to be “experiential experts” (El Mallah, 2024, p. 984) on racial disparities in reading outcomes and paid them for their time. At the time of the research, the community members were 46% women of color, 38% White women, and 15% men of color. The 3Rs university staff were predominantly women (93%), the majority were White (57%), and about one‐third identified as women of color (36%). In comparison to the general population of the county, the 3Rs Team overrepresented women and people of color.

The 3Rs Team met monthly to explore new project ideas, ask questions, review updates, and make decisions. The members strived to hold one other accountable to 3Rs' values, particularly the initiative's racial equity tenet by keeping the group focused on actionable ideas that benefit Black children and families. These meetings reflected the kind of space where people could show up authentically and speak truths with love and presence (Wanless et al., 2023). They were together each month for over a year—through the first year of the COVID‐19 pandemic, the public murders of Ahmaud Arbery, Breonna Taylor, George Floyd, and other Black citizens, and the heightened public reckoning with racial injustice. As such, they came to understand that the 3Rs team was more than a committee; it was a community committed to relationship building and racial equity within its own functioning and across the county. As one community champion said, “I need this [3Rs Team] in my life. I need to know I'm not by myself as I struggle to undo what I always understood as truth.”

The 3Rs Initiative's commitment to CBPR was essential because past efforts had been driven by researchers or by community leaders, but never as a partnership that blended the strengths of both groups. As the 3Rs research team grew to appreciate the constellation of educational partners, community organizations, and families that guided children's learning, it was evident that a CBPR approach would lend itself to an ecosystem initiative that included many perspectives on the issue. As was evident in the 3Rs Initiative, CBPR also had the added benefits of building trust between academic institutions and communities (Flicker et al., 2010; Gonzalez & Trickett, 2014).

## STUDY 1

### Study goals

Although improving reading scores and decreasing racial gaps in reading were the goals, the 3Rs ecosystem‐wide programming was working toward those goals by changing adult knowledge and beliefs. To hold itself accountable for immediate outcomes, a definition (and later a measure) of adult 3Rs mindset was needed. The aim of Study 1 was to co‐construct a definition of the 3Rs mindset to ensure the integration of reading, racial equity, and relationships was informed by community and university expertise. Processes for defining constructs are well‐documented in the literature (DeVellis & Thorpe, 2022; Furr, 2018; Zhou, 2019), but processes for co‐defining constructs with community members using CBPR have received less attention (Arora et al., 2015). To be valid, the 3Rs mindset definition would need to center community wisdom, reflect an unwavering commitment to racial equity, and be informed by extant research. To be practical, the definition would need to be easy to understand and apply by any adult in the community, no matter their role or background. Participatory qualitative analysis, a process that involves community members in data interpretation (Burgess et al., 2021), was the optimal approach for collaborating with diverse community members to co‐construct a contextually specific definition (Gonzalez & Trickett, 2014; Vinokurov et al., 2007).

### CBPR process

Co‐defining the 3Rs mindset occurred in five steps from August 2019 to November 2020, with the last being analysis (Figure 1). First, the 3Rs staff conducted 22 interviews with 30 community and university experts about their thoughts on the state of reading, racial equity, and relationships in the local literacy ecosystem. Seventeen of the interviews were with individual people, and five interviews were with someone who invited between 1 and 4 additional people that they worked with to join them in the interview. Of the 30 people who were included in the 22 interviews, 18 were white women, 7 women of color, 3 men of color, and 2 women without other demographic information available to report. They represented a wide range of roles throughout the literacy ecosystem (and many held multiple roles), including parent, grandparent, activist, family support center staff, public school educator or administrator, school board member, charter school educator or administrator, literacy organization volunteer or staff, researcher, and preservice teacher education faculty. These experts were identified by the 3Rs Team members, who collectively had a long history of working across sectors and neighborhoods in the county. At the end of each interview, we asked who else we should be speaking with. This question generated new names via snowball sampling that eventually reached saturation.

Second, two focus groups were conducted with 13 staff and leaders from local literacy organizations. Some were identified through the initial interviews, while others were recruited via word‐of‐mouth and overlapped highly with main literacy convening groups in the community, including one facilitated by the United Way of Southwestern PA. The organizations varied in size, demographic served, and longevity, and were representative of the larger literacy ecosystem, which was mapped in a more comprehensive report developed at the beginning of this project (Orman et al., 2021). These focus groups provided an organizational perspective. In the first focus group, organizational representatives were provided a local newspaper article (Sheehan, 2019) and report (A+ Schools, 2018) that outlined racial disparities in the local literacy ecosystem, coupled with parent quotes about their experiences (e.g., “When I asked his teacher about my child struggling in school, she blamed me and said that it is because parents don't read with their kids or help them with homework”). The article, report, and quotes were chosen to provide different perspectives on the current state of the literacy ecosystem, and to elevate marginalized perspectives. Specifically, the newspaper article was chosen because it summarized a number of recent efforts to call attention to ongoing racial disparities in the school district, driven by a Black school board member who was a respected elder in an under‐resourced, African American neighborhood. The report was written by a nonprofit organization initially created in response to recommendations by a mayor's commission in 2003. It summarized data, by race, to let the public know how the schools were serving children and families, with comparisons to past years and to cities that were comparable on key indicators. We shared findings from the report that were specifically about literacy. Finally, four quotes from families highlighted their concerns and exasperations in their own words. This structure of multiple local data sources enacted our team's values by highlighting data, voices of a respected Black community leader, and local families, instead of the voices of the focus group facilitators, who were university employees. Focus group facilitators were familiar with the source material and were part of the 3Rs Team that had spent months meeting monthly and discussing the literacy ecosystem. Representatives were asked to reflect on these documents and what they wished for literacy in the county. In the second focus groups, representatives were asked to envision what it would take for the county to start incorporating all three Rs in its approach to early literacy learning.

Third, the 3Rs cohort of community members was asked, in a meeting, to describe what the county would look like in 10 years if it were “3Rs‐ish.” Specifically, what would “3Rs‐ish” adults and organizations be doing that would demonstrate they were integrating all three R's? Finally, one of the 3Rs co‐leaders interviewed 11 3Rs staff about what it might look like for a person, organization, or county to integrate each of the three Rs and become “3Rs‐ish.” Staff were also asked if they knew anyone who was doing this now.

### Key analytic steps and strategies to ensure credibility in findings

In this study, we addressed trustworthiness by establishing credibility (Ahmed, 2024). Credibility is the extent to which our findings reflect participants' realities. One way to strengthen credibility is prolonged engagement in the community and building rapport with participants. From the beginning, the office that houses the 3Rs Initiative was selected because it had over 35 years of engagement in the community, with strong rapport and networks that are particularly dense in marginalized neighborhoods. During Study 1 in particular, the 3Rs Team spent time participating in community meetings held by existing literacy networks, leading and observing nine literacy professional development sessions in schools that struggle most with racial disparities in literacy scores, and engaging in large, interactive, countywide quarterly community gatherings held by the 3Rs Initiative's parent study. The 3Rs Team also spent considerable time engaging in the history of the literacy ecosystem through document review and conversations with people who were part of those historical milestones (e.g., the Beginning with Books initiative, the Triple M program, reviewed a report and heard testimony from a lead advocate in the 20‐year history of a lawsuit against the largest public school district for racial inequities, reviewed a book by Barbara Sizemore who was a local advocate for racial equity in education, etc.). All these activities occurred outside of the formal data collection efforts included in Study 1, but they set the stage for prolonged engagement that built trust, rapport, and perspective for data analysis.

Study 1 also aimed to bolster trustworthiness through triangulation, by using multiple data collection methods and sources to examine the same phenomenon: An adult 3Rs mindset. These included semi‐structured ethnographic interviews with key informants, structured focus groups with literacy organization staff that reacted to key community artifacts from multiple perspectives, a focus group discussion with the 3Rs Team's vision for the literacy ecosystem, and individual interviews with staff working on the 3Rs Initiative. The data from each of these sources was combined into one data corpus that was analyzed as a whole. Quotes from each source were labeled for their source; however, to ensure that domains and subcomponents were represented across themes (and not just coming from one source).

Finally, to limit biases or perspectives of the analysts, findings were shared with the 3Rs team (university and community members) for debriefing and critique. When any uncertainty about codes remained, our group with diverse backgrounds and roles shared decision‐making and came to consensus. This debriefing strategy aimed to increase the confirmability, and thus trustworthiness of the findings.

Transcripts from all steps (i.e., external interviews, organization focus groups, meeting notes, and staff interviews) were coded by 3Rs staff using reflexive thematic analysis. The subjectivity inherent in the process leveraged the coders' in‐depth contextual knowledge but also required them to continuously reflect on their positionalities as White women associated with the university (Braun & Clarke, 2022; Nowell et al., 2017). The 3Rs Team was led by a White woman and a Black woman, and a White woman was the project manager. They met frequently to discuss the team's work, direction, decisions, and to debrief what they learned. The Black woman had extensive experience dealing with racial inequities in her own life, facing racial inequities in a wide range of schooling systems, and studying racial inequities in her professional role. Her ongoing reflective conversations with the White women (who coded the data) played a major role in helping to keep racial positionality at the forefront (Kellam & Cirell, 2018). In addition, the 3Rs Initiative's parent study (The Pittsburgh Study) offered sessions on antiracism for university and community study members. These sessions surfaced issues of racial positionality and power. Finally, the 3Rs Initiative's office (The Office of Child Development) and school (The School of Education) each had monthly staff trainings on antiracism. One was specifically for “White Co‐Conspirators” and was led by the lead analyst in this study. Given the critical importance of race in this study's topic and in the time and place of the study, self‐reflection and self‐monitoring were prioritized to remain vigilant about the ever‐present biases that White women can have (Kellam & Cirell, 2018).

They read all transcripts twice before inductively and iteratively generating codes in subsequent readings (Saldana, 2021). Specifically, potential themes were constructed in the first cycle of coding. Concept codes were created to reflect the meaning of the quote. These concept codes were intentionally broad in the first cycle of coding and ultimately became the four domains of the definition. Commonalities emerged because the domains tapped distinct issues that cut across data sources. The second cycle of coding occurred within each of the domains, to generate subcodes that ultimately became the domain's subcomponents. The data that were initially sorted into a domain were re‐read and coded with more specificity. When codes became domain names and subcodes became subcomponents, words were added for clarity and richness. A review of the subcomponents suggested that there was sufficient coherence within domains. Indeed, during the review conversation with the 3Rs Team, there were no questions about the placement of subcomponents within domains, and no suggestions that subcomponents may be better placed elsewhere.

It was important that the 3Rs mindset definition could apply to all adults in the literacy ecosystem, so any comments that were focused on one role (e.g., what families should do, what teachers should do) were grouped together across roles based on the underlying idea. An example of this is the domain, “Working Together” that combines sentiments that educators should reach out to families more and that families should not have to feel so isolated in figuring out how to teach their children literacy skills. During the data reduction process, quotes were sorted into themes and sub‐components defining a 3Rs mindset and were brought to the 3Rs Team meeting for review and interpretation. The group was asked to provide reactions, and to name the most exciting part of the definition, parts of the definition they would change, to describe what they envisioned a county truly embracing the definition would look like, and to determine how well the definition captured the spirit of the 3Rs mindset. Coding and validation were divided so 3Rs staff could do the time‐intensive coding, while community expertise could be leveraged to revise, refine, interpret, and validate the final definition (Israel et al., 2005).

### Results

The 3Rs mindset was defined with four domains: (1) Loving Black Children, (2) Embracing Literacy, (3) Prioritizing High‐Quality, Racially Affirming Books, and (4) Working Together (see Table 1). Each domain included 3‐6 sub‐components. The 3Rs Team interpreted the 3Rs mindset as a vision incorporating the values and hopes of a community. After the coding, revision, and validation process, 3Rs Team members (*n* = 10) were asked, “Overall, how much does this definition capture the spirit of the 3Rs?” On a scale of 1–5 (1=does not capture, 5=full captures), members rated it highly (*M* = 4.7).

**Table 1:** Co‐constructed definition of a 3Rs mindset.

Domain	Definition	Sub‐components
Loving Black Children	Adults see strengths in Black children and families and love them like their own.	(a) Celebrate Black children's autonomy rather than compliance. They stop policing Black children's behavior. (b) Advocate for Black children and against all racial injustice. (c) Know that every family is literate in their own way, including the use of African American Vernacular English. (d) Honor Black culture & communities. (e) Act on the knowledge that Black families are assets to children's development. (f) Maintain high expectations for Black children.
Embracing Literacy	Adults are always engaging children in literacy experiences.	(a) Literacy includes conversations, writing, print, books, and storytelling. (b) Adults and children often feel joy when reading together. (c) Children see adults loving literacy. (d) Child literacy is everyone's goal, not only higher standardized test scores.
Prioritizing High‐Quality Racially Affirming Books	Our community is full of adults who are effectively using books and materials that affirm Black culture.	These books are: (a) Mirrors & Windows. (b) Show Black characters with agency. (c) Convey to Black children that books can help them reach their dreams. Adults use these books to: (d) Have honest, age‐appropriate conversations with children around racial equity. (e) Help children feel empowered to disrupt racial equity.
Working Together	Adults and organizations work together so everyone has the support and resources they need and can align their efforts.	(a) Teachers and families have strong relationships and view each other as trusted partners. (b) Schools, libraires, and organizations align their efforts for greater impact. (c) Shared decision‐making for creating projects, spaces, and materials that reflect the community and proactively seek out Black voices at the table.

Loving Black Children, one domain, reflected an asset‐based mindset that honored and advocated for Black children, culture, and communities in literacy and beyond. One comment from a parent and literacy researcher represented this sentiment: “When we do not portray all families as literate in their own way, we do a racial injustice. Yet it happens all of the time.” The 3Rs cohort of community members said this domain was foundational to the rest of the definition and was what set 3Rs apart from previous initiatives. They emphasized that students have a right to show up in their own language and that African American Vernacular English is a language. That was then added to the definition. Finally, there was ongoing discussion around what advocacy meant, including speaking up outside of 3Rs team meetings. Many also expressed that Loving Black Children was the most exciting part of the definition, as this was the piece most commonly missing in previous local initiatives. Within this domain, they discussed having high expectations for Black children as essential. As one person from the 3Rs cohort of community members noted, “Children live up to what the adults in their lives expect of them. So, expect the maximum and they will stand and deliver!”

Embracing Literacy reflected a mindset that prioritized engagement in joyful, multimodal literacy experiences with children that are age appropriate and engender holistic literacy success. One 3Rs staff member expressed this need for a holistic view when she said she wished everyone saw, “learning as more than just test scores.” Many expressed a need for more joy in literacy experiences, and parents described how bored children were during school literacy time. One parent said, “My son will do the work that they tell him to do without asking for more, and even he got bored by the end of pre‐K. My daughter has a thirst for learning. What will happen to her?” A local children's librarian summed it up well by saying, “I think that enjoying reading is what makes kids stick with reading.” Insights from the 3Rs cohort of community members included wanting to emphasize love and joy in reading and literacy and shifting focus from teachers to encompassing all adults in the literacy ecosystem. As a result, the word “joy” was added, and two subcomponents that were specifically about teachers (“teachers know and intentionally teach to children's reading level,” and “having sustained professional development opportunities and sufficient planning time for literacy”) were deleted because they did not apply to all adults in the literacy ecosystem.

Prioritizing High‐Quality, Racially Affirming Books reflected a mindset that valued books that affirmed Black identity and culture for children and families. One mother and education advocate stated, “I never heard of a list of books for African American…boys. How are they supposed to stay interested…when none of it is about them?” When creating this domain, they emphasized that it was important to explicitly state that it was not just about increasing access to books, but also about how adults used the books skillfully. One literacy researcher said that using books skillfully included offering them as “windows and mirrors for all kids, and ways of being Black.” When the 3Rs Team reviewed this domain, they added two subcomponents about adults using books skillfully, to be clear that just having high‐quality, racially affirming books was not enough. These subcomponents reflected many conversations the 3Rs Team had had in their past meetings.

Working Together reflected a mindset that recognized the necessity of cross‐sector networking, resource sharing, and decision‐making to ensure children and families were embedded in an ecosystem of literacy support. One quote from a grandparent and community outreach worker was representative of many similar statements: “You are not going to change reading scores just by reaching the kids. You have to reach the parents. Parents are sometimes very hard to reach. Single parents are even harder. Single parents who work several jobs are very hard to reach. But if you want to reach them, you can.” Literacy organization staff called for a different approach to working with families. “We don't need to blame parents. We need to support them. When kids can't read, parents already feel like a failure.” In the focus group with literacy organizations, there was agreement that making decisions without having families at the table is common and problematic. “We cannot and should not assume that all parents think like educators.” The 3Rs cohort of community members wanted to shift the focus from “welcoming everyone's voices” to asserting that predominately White organizations need to proactively seek and make space for Black voices at the table. That edit was made during the 3Rs Team's validation discussion.

### CBPR process outcomes

Process outcomes indicate how the collaborative research context (e.g., partnerships with communities) has been transformed through the CBPR process (Sandoval et al., 2012). In Study 1, the primary process outcome of co‐defining the 3Rs mindset in the 3Rs Team was the mutual strengthening of the community members' and researchers' resolve and optimism. The 3Rs mindset definition was co‐constructed by first listening to and elevating community voices, then by blending community and researcher voices, and finally by seeking validation from the 3Rs cohort of community members. The co‐constructing process made the community members feel, “excited, hopeful, proud, like the work has finally begun” and “At peace; so hopeful; like we are finally doing right by more of our children.” One reflected, “The community [member] role reminds me of the quote: ‘Nothing about us, without us, is for us.’” However, they also kept the research team grounded by reminding that the 3Rs mindset was not a panacea: “Many more aspects of life in [our county] must also be transformed to picture a 3Rs future.” Partnering with community members provided researchers with a sense of humility and pride and a sense of purpose to make sure the 3Rs mindset could be measured. The researchers knew that a measure would be needed to keep them accountable for the impact of any of the 3Rs Initiative's future work, and to attain funding for it. The 3Rs cohort of community members agreed that measurement was important, and that decision led to Study 2.

## STUDY 2

### Materials and methods

Study 2 aimed to co‐construct a practical and psychometrically sound measure of the 3Rs mindset for any adult in the literacy ecosystem (e.g., teacher, librarian, grandparent, nonprofit director). Creating one measure for any adult was important because adults served multiple roles and sometimes changed over time. The 3Rs Team (university staff and a cohort of community members) collaborated for 18 months to conceptualize, design, develop, and validate the 3Rs Mindset Scale, or 3Rs‐MS. While some traditional measure development processes emphasize the importance of community input to ensure construct validity (DeVellis, 2012), this often takes place only during the pilot phases (Flicker et al., 2010). Collaborative survey development, on the other hand, prioritizes community engagement through the entire research process (Arora et al., 2015). In the present study, the 3Rs Team utilized the three tenets of CBPR (collaboration, honoring multiple perspectives, and prioritization of social change; Strand et al., 2003) within a framework of collaborative survey development (Flicker et al., 2010; Schulz et al., 2005).

As shown in Figure 1, the creation and validation of the 3Rs‐MS occurred in five phases from August 2020 to February 2022. In the first phase, the 3Rs Team met monthly to discuss the purpose and optimal format for the 3Rs mindset measure. They raised concerns about asking directly about people's racial equity mindsets because some may answer with what is expected instead of how they actually feel (social desirability; Paulhus, 2002). During this time, there was a lot of discussion about racial equity across the country, particularly in response to a number of killings of Black people that were highly visible in the national media. The 3Rs Team felt that people were highly aware of how they “should be” responding to questions about racial equity, and wanted a way to limit that in the data. The 3Rs Team researchers explored measurement approaches and found past literature about using vignettes as a way to limit the effects of social desirability (e.g., Hughes & Huby, 2012). After sharing a review of the literature on measurement development with the rest of the 3Rs Team, and examining example vignettes from a study that aligned with 3Rs measurement goals (Gilliam et al., 2016), they decided to use vignettes to avoid social desirability and cognitive bias in respondents (Evans et al., 2015; Stecher et al., 2006). They also decided it was important use African American sounding names in the vignettes to reflect implicit biases. The 3Rs Team members repeatedly emphasized the need to keep the 3Rs definition's focus on Black children in the measure. As one community member said:

Everyone will affirm literacy. “Yes, I read to my child. Yes, I think that reading to children is important.” I think that the questions of, “Do you think that it is important to have books that affirm Black children's identity, and do you believe that Black children have their own language that is valuable” are the key points.

In the second phase, 18 short vignettes were created (2–5 sentences each), one for each subcomponent in the 3Rs mindset definition. Each vignette was followed by three questions (two positively worded and one negatively worded) intending to tap into different aspects of the subcomponent. Response options were a Likert scale ranging from 1 (strongly disagree) to 5 (strongly agree), with reverse scoring for negatively worded items. Scores were calculated as the average score across all 54 items on the scale.

In the third phase, the short vignettes were subjected to rigorous review by the 3Rs Team, for content validity (El Mallah, 2024). Initial reviews led to critical revisions regarding survey wording and refining vignettes to match the intended 3Rs mindset domains and subcomponent(s). In a final meeting, the 3Rs Team conducted a review of scale items to ensure they reflected all four domains and 18 sub‐components of the 3Rs mindset definition.

In the fourth phase (September 2021–February 2022), the 3Rs university staff administered the first pilot test of the 3Rs‐MS to two separate groups. The first group (*n* = 17) included five individuals from the community who had been identified as potentially having a strong 3Rs mindset and 12 additional adults from the literacy ecosystem who were unfamiliar with the 3Rs project (e.g., teacher, author, psychologist, nonprofit executive, parent). Researchers conducted cognitive interviews (Knafl et al., 2007) while they were taking the 3Rs‐MS. The first five were theorized to have strong 3Rs mindsets and were interviewed for content validity (i.e., the extent to which items on the 3Rs‐MS reflected the 3Rs mindset definition; DeVellis & Thorpe, 2022), while cognitive interviews with all participants provided evidence of response process validity (i.e., the degree to which respondents used the intended psychological processes; Furr, 2018). The second group of pilot test respondents (*n* = 61) was sampled from one of the 3Rs target populations—school personnel—and included teachers, paraprofessionals, reading specialists, librarians, and principals across five schools who took the survey independently to provide quantitative data for convergent validity (17% Black, 83% White; 85% female, 15% male; 62% <45 years old; 71% attended graduate school).

In the fifth phase, data from the pilot tests were analyzed to evaluate psychometrics. While the scale had excellent psychometric properties (i.e., internal consistency and associative validity), participants in the cognitive interviews repeatedly commented that the scale was too long and tiresome. Data from the second group of participants confirmed this, with a clear rise in missing data in the second half of the survey. Using information from the cognitive interviews and initial pilot test, researchers reduced the scale from 54 to 36 items, removing one positively worded item from each vignette. Item‐level decisions were made to reduce content redundancy, to maintain items that produced the most variability among general respondents, and to limit items that had higher variability among the five people who were nominated for their strong 3Rs mindsets. Specifically, researchers examined data from the two pilot samples (*n* = 17, *n* = 61), to decide how to shorten the scale. All of the items were re‐read and their standard deviations and correlations from each sample were reviewed. Three criteria were considered: (1) whether any items seemed redundant (high correlations with each other in each sample, and the wording looked like it was asking about highly similar content), (2) whether any items showed little variability in the pilot sample from school personnel (low standard deviations) and (3) whether any items showed high variability among the five people who were nominated for their strong 3Rs Mindsets (high standard deviations). Low variability was considered an indicator that this item was not helping to differentiate respondents and high variability among the people with strong 3Rs Mindsets indicated that the item may not be consistently tapping into the idea it was intended to reflect. In fact, after reviewing the cognitive interviewing transcripts for those items with high variability for people with strong 3Rs Mindsets, it became clear that they were each interpreting the items somewhat differently.

Through this review, it was evident that the items having the most concerns (content redundancy, low variability for school personnel, and high variability among people who strong 3Rs Mindsets) were always one of the positively worded items for each vignette. Therefore, the positively worded item that was most problematic, from each vignette, was removed. Any other items that showed issues according to the three criteria, were reviewed and the cognitive interviewing notes associated with the items were re‐read. In cases where one word or detail seemed to be moving the respondent away from the intent of the item, that detail was removed or changed for clarity. Researchers then conducted psychometric analyses on the 36‐item scale with a second group of school personnel (*n* = 52; 41% Black, 59% White; 87% female, 13% male; 76% <45 years old; 71% attended graduate school).

### Analytic strategy

Analysis focused on three dimensions of construct validity: content, response process, and convergent association (Furr, 2018). Content validity included domain representation (i.e., are all domains present in the scale), domain relevance (i.e., are all scale items relevant to the domains), and test construction (i.e., did the process of creating the scale reflect contextually and culturally relevant practices) (Almanasreh et al., 2019). Domain representation and domain relevance were addressed concurrently by having the 3Rs Team members independently, collectively, and iteratively reviewed the scale to ensure all domains were captured and all items reflective the intended domains. Test construction was assessed by examining contextual and cultural relevance based on 3Rs Team discussions and cognitive interviews. Response process validity was examined by reviewing cognitive interviews for themes in interpretation and thought processes (Padilla & Benítez, 2014).

After establishing content and response process validity, associative validity was analyzed by correlating 3Rs‐MS scores with individuals' scores on several related scales. The related scales all showed acceptable to good internal reliability and included racial humility (adapted from Wang et al., 2003; 4 items, *α* = .83), knowledge related to children's racial identity development (based on framework in White & Wanless, 2019; 4 items, *α* = .79), attitudes towards systemic racism/colorblindness (adapted from Bento & Brown, 2021 and Neville et al., 2000; 6 items, *α* = .84), and beliefs about how teachers relationships impact learning (adapted from Hawley et al., 2021 and Kim et al., 2015; 9 items, *α* = .67). Finally, reliability of the entire 3Rs‐MS as well as each of the four 3Rs domains were assessed using Cronbach's alpha.

## RESULTS

The average score on the 3Rs‐MS was 4.00 out of 5.00 (SD = 0.54). There were no significant differences in 3Rs‐MS scores by age (*t*(49) = 0.006, *p* = .996), gender (*t*(50) = 1.75, *p* = .09), race (*t*(47) = 0.10, *p* = .92), or educational background (*t*(50) = 0.81, *p* =.42). For reliability, Cronbach's alpha for all items on the 3Rs‐MS was 0.94, reflecting excellent internal consistency (Furr, 2018). Cronbach's alpha for items within each domain ranged from good (0.75 for Working Together and 0.77 for Embracing Literacy) to very good (0.82 for High‐Quality Racially Affirming Books and 0.87 for Loving Black Children).

### Content validity

Joint assessment of domain representation and domain relevance revealed that—after iterative revisions—the items comprehensively captured the domains. Feedback included connecting items to the 3Rs mindset sub‐components by using language explicitly tied to the Study 1 definition rather than veiled language. For example, for a vignette about prioritizing books that depict Black characters with agency, rather than say, a Black character in the book “dreamed about this career and made it happen despite many people suggesting that they choose a different career,” a community member recommended directly saying the character “overcame discrimination throughout their career.” Additionally, it was suggested that using either/or language was sometimes inappropriate. For example, a vignette about celebrating Black children's autonomy initially seemed to suggest that one could either respect Black children's autonomy or have high expectations for Black children, but not both. The item was revised to reflect having high expectations for Black children while also respecting their autonomy.

Finally, construct irrelevance was reduced by highlighting unnecessary vignette and item details (e.g., removing the words “reading level” from vignettes and items when reading level was not relevant to the 3Rs mindset component). No evidence of construct underrepresentation was found; each component of the definition was reflected in the scale.

### Response process validity

In cognitive interviews, it was clear that vignettes and items needed additional demographic information to limit assumptions, such as stating the race and role of the adults and the child's age. 3Rs community members recognized that race was only described when vignette characters were not White, which was a form of racialized scale creation and was addressed. Also, respondents grappled with their own positionality and racial lenses while taking the survey. For example, one participant said that learning about harmful racialized reading practices over the last year impacted their response choice to a question about representation in children's books. Others pointed out that the negatively worded items reflected a White racial lens, which they were intended to because the opposite (i.e., positively worded) of the White racial lens would be a 3Rs mindset. This provides evidence of response process, as identifying and responding to racial representation is a key aspect of the 3Rs mindset.

Another theme from the cognitive interviews was tension between how respondents felt and the rules in local systems and organizations. For example, two participants referred to structural rules (e.g., “the rules [in school] say we must do…”) when selecting their response choices, indicating the rules contrasted with their own mindset. This only happened with two participants and on a minority of the questions. Nonetheless, this highlights the importance of scale instructions, encouraging respondents to answer how they feel and not “how things are.”

### Convergent validity

The 3Rs‐MS scores were positively significantly correlated with scores on the racial humility scale (*r*(48) = 0.48, *p* < .001), positive racial identity scale (*r*(48) = 0.34, *p* = .02), attitudes towards systemic racism and colorblindness scale (*r*(47) = 0.49, *p* < .001), and beliefs about how teachers' relationships impact learning (*r*(48) = 0.57, *p* < .001).

## DISCUSSION

This paper offers an illustration of how CBPR tenets can be applied in a construct and measurement development process, specifically within a community‐university partnership. As community‐engaged research gains traction in higher education, there is a growing need to blend university and local community expertise—not only to define what issues matter, but also to collaboratively determine how they should be measured (Ohmer et al., 2022). However, published examples of truly collaborative measurement development remain limited, often emerging in specific contexts such as work with Indigenous communities or Youth Participatory Action Research (e.g., Aldana et al., 2019; Gonzalez & Trickett, 2014). Yet, designing culturally sensitive and contextually grounded measures is critical to both validity and equity. Measures must reflect the cultural realities of the communities where they are developed and used. As El Mallah (2024) writes:

Racially and ethnically diverse populations from minoritized backgrounds are often exposed to research methodologies that amplify structural racism and negate their sociocultural reality. Although cross‐cultural validation of measures is considered a requisite step to multigroup comparisons, researchers apply measures validated and standardized in the dominant White culture to under‐researched populations (without assessing measurement equivalence first). (p. 978)

This underscores the need for research approaches—like the one described here— that prioritize cultural validity from the outset through deep community involvement in construct definition and measurement design.

This imperative to ground measurement in community realities led the 3Rs Team to move beyond adapting existing tools. Rather than reshaping dominant‐culture constructs to approximate community needs, we co‐created new ones rooted in local priorities and cultural specificity. In the present study, the 3Rs Team faced a reality that decades of literacy programming were not serving Black children well. In addition, existing constructs and measures had not been developed to reflect the needs and experiences of Black children in this community. To fully express the need to shift more power toward families, community leaders, and elders who have been advocating for Black children for years, we created a space where a new vision for the future of the literacy ecosystem could be co‐developed and measured. We did not try to adapt an existing construct and measure to more closely match the outcome that the 3Rs Team aspired to. Instead, we let the 3Rs Team's outcome stand on its own. Similar to Gonzalez & Trickett's (2014) description of their 3‐year collaborative measurement development. process, “the commitment to the collaborative process across multiple levels of…input generated a greater trust in and local support for the work.” (p 18).

In Study 1 and Study 2, we offer an example of this process that was iterative, took time, and honored the history of the place we were in. Specifically, in Study 1, the 3Rs Team used participatory qualitative analysis to engage community and university experts across multiple sectors to co‐construct a 3Rs mindset definition that was culturally valid and addressed a community‐identified issue (i.e., racial inequities in early literacy). The result was a culturally and contextually valid definition of a 3Rs adult mindset with four domains: (1) Loving Black Children, (2) Embracing Literacy, (3) Prioritizing High‐Quality, Racially Affirming Books, and (4) Working Together. In Study 2, the research team co‐constructed the 3Rs‐MS measure by integrating the CBRP principles of collaboration, honoring multiple perspectives, and social change (Strand et al., 2003) with an expansive, approach to measure development and validation (El Mallah, 2024). Findings from Study 2 indicate that the 3Rs‐MS reliably captured the four 3Rs mindset domains and had evidence of construct validity.

In addition to these findings, one of the key takeaways is that the sequential design from Study 1 [construct definition] to Study 2 [measure development] was not predetermined from the outset by researchers, as is more common in measure development (DeVellis & Thorpe, 2022). Rather, it emerged organically from the 3Rs Teams' engagement with the community over time. This reflects what is known about CBPR research, namely that it “provides an inclusive and flexible research framework” that allows for more community‐centered, “transformative and pragmatic approaches to the research process” (Collins et al., 2018, pp. 2–3). It is possible that our work with the community could have taken us in any number of directions (e.g., advocacy, classroom‐based interventions, etc.), which may not have led us to the 3Rs mindset and measure. The flexibility CBPR affords, therefore, allowed us to respond to the community's needs in real‐time while also improving the validity of our findings and establishing the sustainability of our initiative (Horowitz et al., 2009), both of which we expand on below.

Our findings also contribute to broader CBPR efforts within community psychology by demonstrating how relational, iterative work can yield both rigorous measures and transformative partnerships (Boness et al., 2025; Gonzalez & Trickett, 2014). The 3Rs initiative illustrates how community‐engaged measurement can advance the field's core commitments to ecological validity, cultural responsiveness, and equity (Trickett, 2009). While CBPR has been widely used in areas such as public health and housing, this study extends its application to literacy and measurement science—areas still emerging in community psychology.

### CBPR tenets and content validity

Content validity is essential to creating measures that capture their intended construct (Delgado‐Rico et al., 2012). In CBPR, content validity has elevated importance because the construct of interest (e.g., 3Rs mindset) and the issue the research is intended to address (e.g., racial inequities in reading outcomes) often reflect cultural and contextual specificity (El Mallah, 2024). The 3Rs‐MS was also anchored in racial equity tenets, and therefore, centering the lived experiences, meaning‐making, and culture of Black community members was central to building the content validity of the 3Rs construct and scale. This extends previous work by demonstrating that including community members can also ensure measures are culturally responsive to those whom the survey is *about* (El Mallah, 2024). This latter finding aligns with Malorni and colleagues' (2023) research demonstrating that including youth of color in the measure development process helped challenge widespread “white dominant epistemologies” (p. 153) often seen in scale development efforts, resulting in the development of constructs and scales that are more structurally and culturally responsive. Collaborating with community members to define the 3Rs mindset and measure was essential to creating a valid scale based on a well‐defined construct rooted in community (Arora et al., 2015). Further, by integrating CBPR principles throughout the process of construct definition and scale development, this study reflects and advances community psychology's long‐standing emphasis on shared power, contextual relevance, and social justice in research (Prilleltensky, 2001; Wallerstein, 2021).

### Slow science with relationship building

Building trusting, reciprocal relationships with community members was the foundation of the present study, but it took years and occurred in an office that had over 35 years of community‐partnered history. The importance of time investment has been well‐documented in previous research on CBPR (Flicker et al., 2010; Strand et al., 2003). As the 3Rs Initiative also addressed racial equity, it required ongoing efforts to continually identify and transform the team's own internal biases. The need to slow down and take time was possible because the lead researcher on the project already had tenure and worked at an institution that had developed promotion guidelines that reflected an awareness of the pace of high‐quality community‐engaged research (Dostilio, 2017). Also, the project had a stable funding stream that allowed for long‐term planning. It was challenging to slow down, however, because everyone was acutely aware of the injustices that were resulting in low reading scores. The need to disrupt this pattern was palpable, but so was the awareness that effective change rarely happens quickly.

The present research joins a growing call for “slow science” (Frith, 2020)—a deliberate, reflective approach to research which “promotes research practices and communities with a high degree of engagement and critical reflexivity, considering the scientific and societal prerequisites as well as the means and outcomes of research” (Salo & Heikkinen, 2018, p. 88). CBPR is a form of slow science that reflects the complexity of the issues it seeks to address, as demonstrated by the 3Rs Initiative. As Flicker et al. (2010) noted, there are no “fast lanes” to high‐quality CBPR; yet CBPR is essential to research aimed at social change (Wallerstein & Duran, 2010). This tension—between the slow, relational work of CBPR and the demand for rapid social change—is at the heart of community psychology. The field has long emphasized not just collaboration, but the importance of sustained, trust‐based relationships as mechanisms for equity and justice (Prilleltensky, 2001; Trickett & Espino, 2004). CBPR contributes procedural clarity to this ethos by offering a framework through which such relationships can generate rigorous and contextually grounded science (Center for Social Justice and Community Action, 2022). In this way, our work illustrates how community psychology and CBPR together hold the dual commitments to both process and impact, honoring the time it takes to do the work well while remaining accountable to the pressing needs of the communities we serve (Rodriguez Espinosa & Verney, 2021; Wallerstein, 2021).

### Affirming all 3Rs and elevating racial equity

Participants' responses in the pilot tests were significantly correlated with their scores on other scales of racial beliefs and knowledge (ranging from 0.34 to 0.49) and a scale of attitudes about how children learn (*r* = 0.57). This provided evidence of convergent validity as a 3Rs mindset is thought to be comprised of attitudes and beliefs about racial equity, relationships, and reading experiences. However, this also demonstrates that none of the other scales fully captured the 3Rs mindset, reinforcing its uniqueness as a construct that blends racial equity and relationships with literacy development. To the best of our knowledge, no other scales cover this unique blend of beliefs and attitudes.

Further, partnering with community members influenced the interpretation of these results by reminding researchers that racial equity—and Loving Black Children specifically—is the foundational tenet that “sets us apart from previous initiatives.” Racially equitable literacy instruction is key to disrupting opportunity gaps in reading outcomes (Spear et al., 2023), and loving Black children is fundamental to racially equitable literacy instruction (Muhammad, 2022). As Love (2019) writes, without loving Black children and celebrating Black joy, equitable teaching is not sustainable. The 3Rs Initiative made racial equity visible, measurable, and nonnegotiable. This emphasis on naming and measuring racial equity aligns with community psychology's goals of dismantling systemic oppression and fostering collective empowerment (Fernández, 2023; Griffith et al., 2007). By co‐creating a construct that explicitly names racial equity, our work contributes to a growing literature within the field that operationalizes equity in ways that are grounded in community voice and action (Rodriguez Espinosa & Verney, 2021; Wallerstein, 2021).

### Implications

This study highlights the potential of CBPR and slow science approaches— rooted in a community psychology framework—to transform the ways we define and measure equity‐focused constructs in literacy and education. For researchers, it underscores the importance of partnering with communities to co‐define constructs, design culturally valid measures, and respond to evolving priorities. Such collaborations can deepen the ecological and cultural validity of measures and shape how educational issues are identified, defined, and addressed. In policy, it suggests a shift toward supporting long‑term, relational, and context‑driven research as a foundation for equity‐focused literacy interventions, as well as investments in curriculum and practice that center relationships and racial equity. In practice, it affirms the centrality of racial equity—and loving Black children—as a core tenet for shaping literacy environments and professional learning for educators. Together, these studies demonstrate how centering community voice can deepen ecological and cultural validity, yielding tools that foster systemic change across literacy ecosystems and other domains of social justice.

## LIMITATIONS AND CONCLUSIONS

The 3Rs mindset definition and measure now lay the foundation for local ecosystem‐wide literacy programming for all children, specifically Black children. This definition and measure can play an important role in helping the 3Rs program to align with the local cultural and contextual literacy ecosystem (Orman et al., 2021) and the 3Rs' parent study, The Pittsburgh Study (Ettinger et al., 2022). The findings from the present studies, however, should be considered in light of limitations. First, although there were strategies used to increase the credibility and trustworthiness of the findings in Study 1 (e.g., prolonged engagement in the community), broader community validation and additional data sources for triangulation would strengthen the findings. Second, in Study 2, the samples for two of the pilot studies were school‐based professionals (e.g., teachers, administrators), but the 3Rs‐MS is intended to be used with any adult in the literacy ecosystem. However, nonschool‐based professionals (e.g., a parent, a non‐profit leader, an author, etc.) did participate in our cognitive interviews, providing rich qualitative data to validate the use of the 3Rs with these populations. Future research can build on this by testing the reliability and validity of the 3Rs‐MS quantitatively with more nonschool‐based professionals like those from our cognitive interviews. Third, although the 3Rs‐MS was reduced from 54 to 36 items, it may still be too long for people to complete, jeopardizing its validity. To this point, larger samples may be recruited to conduct confirmatory factor analysis to verify the four domains of the 3Rs‐MS. If verified, the measure could be broken down into four sub‐scales, one for each domain. At that point, item response theory and methods could be used to assess which items within each domain are the strongest and weakest in terms of difficulty, discrimination, and false positives (DeVellis & Thorpe, 2022). Expert review—including both academic and community experts—could then interpret these results and recommend items to be retained or eliminated, ensuring each sub‐domain has at least three items so internal consistency can be measured (Furr, 2018). If the confirmatory factor analysis reveals the domains are not a good fit for the data, then the 3Rs Team could explore alternative domains using exploratory factor analyses with a new sample. Caution would be needed here; however, as the four domains remain practically important in terms of representing community voice. Finally, as is typical in community‐based research, these results may not generalize to all literacy ecosystems. We encourage the use of community‐based research across settings. The pacing and step‐by‐step process described in this article may inform that work. Taken together, balancing community experiences and priorities with rigorous psychometric evaluation provides an innovative blueprint for defining and measuring psychological constructs and integrating best practices in CBPR and measure development.
